# Situation of type specimens of *Cordyceps* and allies described by Dr Kobayasi

**DOI:** 10.1080/21501203.2017.1394392

**Published:** 2017-11-07

**Authors:** Hiroki Sato

**Affiliations:** Forest Entomology, Forestry and Forest Products Research Institute, Tsukuba, Japan

**Keywords:** Authentic specimen, cicada, colour illustration, *Elaphomyces*, holotype, lectotype, paratype, *Torrubiella*

## Abstract

Information about authentic specimens of *Cordyceps* spp. that were described by Dr Kobayasi were summarised. Dr Kobayasi, National Museum of Nature and Science, has described/proposed about 28% of the scientific names for the major two genera of entomopathogenic fungi (*Cordyceps* s. l. and *Torrubiella* s. l.) with Mr Shimizu before 2007. In total 44 authentic specimens were discovered at present: 19 in *Torrubiella*, 6 in *Cordyceps* spp. on *Elaphomyces*, 19 in *Cordyceps* spp. on cicada. Although the colour iconography books published by Dr Kobayasi and Mr Shimizu did not explain the information about the illustrated specimens, 22 among the discovered specimens have been illustrated in colour showing their fresh condition in the books.

## Introduction

*Cordyceps* Forum 2016, which was held in Pinghu, provided a platform for crossover discussion on studies of *Cordyceps*. For studies on both the basics and the applications, the most important knowledge is the exact identification of the specimen(s). Preferably, the study specimens are compared with the type specimen for the identification. However, it is sometimes difficult to gain access to the specimens.

Dr Kobayasi (1907–1993), the Director of the Department of Botany in the National Museum of Nature and Science in Tokyo, has described more than 150 species of *Cordyceps* and its allies with the help of Mr Shimizu (1915–1998) who has excellent skills in collecting *Cordyceps* specimens. They mentioned that the type specimens were deposited in the herbarium of the museum. However, because of unfortunate situations, both of specimens (without accession numbers) and of the managing system (fund, space, human resources, data base, etc.), most of those specimens became untraceable. Since 2004, a reordering of the specimens has been conducted. Unregistered specimens scattered in several different lockers were gathered in one room. Several specimens were recognised as type candidates. Some of the specimens were identical to the colour illustrations in the books on *Cordyceps* spp. (Kobayasi and Shimizu 1983; Shimizu 1994). Unfortunately, no information regarding the illustrated specimens (even if it is a holotype or not) was described in the two books.

Some holotype specimens of *Cordyceps* on cicada had been deposited in a private museum of cicada founded by Dr Masayo Kato (1898–1967). He was a pioneer of the cicada studies in Japan and studied not only cicada taxonomy but also the natural enemies of cicadas, including *Cordyceps*. He asked Dr Kobayasi to identify *Cordyceps* specimens that were sometimes new species. After his death, the museum was closed, and all the specimens have been kept by his family. In 2010, all the specimens, including several *Cordyceps* specimens, were deposited by his granddaughter at the museum in the University of Tokyo.

A special effort to identify the types in the National Museum of Nature and Science was initiated since 2005. In addition, I had a chance to check the *Cordyceps* specimens in the museum of the University of Tokyo in 2013. The situation of the type specimens of *Torrubiella* and *Cordyceps* on cicadas and on *Elaphomyces* has been reported (Sato et al. 2010a, 2010b, 2012, 2014). Though *Cordyceps* type specimens from other host groups remain to be rediscovered, listing up the type specimens that are found is useful for taxonomy research. In this manuscript, I have prepared a list of the type specimens as a halfway report.

## Scientific name proposed by Dr Kobayasi

Dr Kobayasi has both described new species and proposed new taxonomic treatments. The scientific names of both the genera *Cordyceps* and *Torrubiella* were sorted in the database “Index Fungorum (http://www.indexfungorum.org/)” during the years from 1801 to 2006. The scientific names proposed after 2007 (including 2007) were omitted because Sung et al. (2007) proposed a new system of taxonomy for the genus *Cordyceps*. The number of species names was counted every 10 years, and the species that were described by Kobayasi were independently counted.

**Figure 1.**
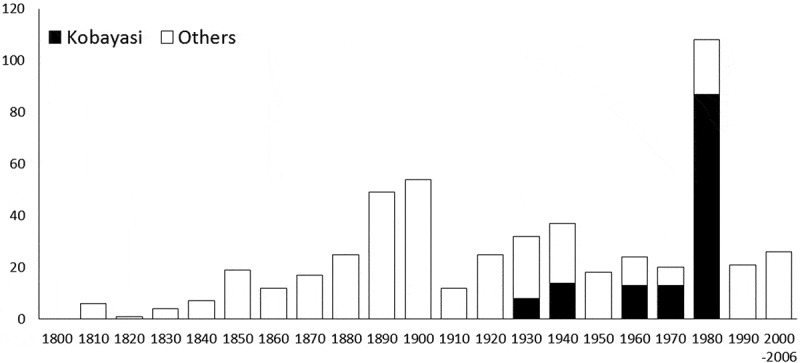
The total species number of every 10 years in *Cordyceps.*

**Figure 2.**
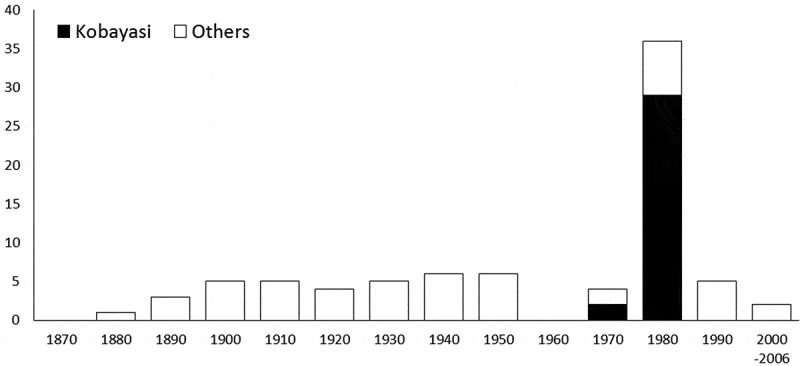
The total species number of every 10 years in *Torrubiella.*

Dr Kobayasi studied *Cordyceps* for about 50 years and has described *Cordyceps* species from 1939 to 1983 (Kobayasi 1939, 1983). His most important publication was Kobayasi (1941), in which he proposed a taxonomic system for global *Cordyceps* spp. Since the 1960s, Dr Kobayasi started to publish manuscripts along with Mr Shimizu who is an excellent collector of *Cordyceps*. Especially, the years between late 1970s and early 1980s were the most active (Figures 1 and 2).

There were 517 scientific names of *Cordyceps* in the Index Fungorum described during 1818–2006 (Scientific names without year information in the database were omitted). In total, 135 *Cordyceps* species name described or proposed by Dr Kobayasi were recognised. About 26% of the scientific names of *Cordyceps* were proposed by Dr Kobayasi, although the total number in the database may have some synonyms or new treatment at present. For the genus *Torrubiella*, he proposed 31 scientific names (including three new combinations), and among the 82 scientific names of *Torrubiella* across the world, about 38% were proposed by Dr Kobayasi. In total, about 28% of the species names for the major two genera of entomopathogenic fungi were proposed by Dr Kobayasi and his collaborator, Mr Shimizu.

## Situation of type specimens

### Genus *Torrubiella*

Dr Kobayasi described 28 species of *Torrubiella*. A total of 19 holotype specimens were discovered. A new name, *Torrubiella plana*, has been given for the species *T. minutissima* Kobayasi, because of homonym problem (Sato et al. 2010a). Information of the type specimens is described in Table 1.

**Table 1.** The list of rediscovered type specimens of *Cordyceps* and *Torrubiella* species described by Dr Kobayasi.

No.	Name by Dr Kobayasi		ID	Status	KS^a^	S^b^	Current name	Literature
	***Torrubiella***							
1	*T.*	*alboglobosa*	TNS-F-12,061	Holo	64–2	352		
2		*aurantia*	TNS-F-12,069	Holo	59–1	330		
3		*corniformis*	TNS-F-12,064	Holo				
4		*elIipsoidea*	TNS-F-12,055	Holo	54–2	345		
5		*formosana*	TNS-F-12,059	Holo				
6		*fusiformis*	TNS-F-234,548	Holo	53–3	360		
7		*globosostipitata*	TNS-F-12,057	Holo	53–2	340		
8		*longissima*	TNS-F-12,071	Holo	64–1	339		
9		*mammiIlata*	TNS-F-12,060	Holo	55–2	-		
10		*minutissima*	TNS-F-12,061	Holo	56–1	347	*Torrubiella plana*	Sato et al. 2010a
11		*miyagiana*	TNS-F-12,062	Holo	56–3	326		
12		*neofusiformis*	TNS-F-12,058	Holo	54–3	323		
13		*oblonga*	TNS-F-12,070	Holo	60–2	322		
14		*ooaniensis*	TNS-F-12,063	Holo	57–1	353		
15		*pallida*	TNS-F-12,789	Holo	58–2	328		
16		*rosea*	TNS-F-12,065	Holo	57–2	317		
17		*ryogamimontana*	TNS-F-12,058	Holo	58–3	341		
18		*ryukyuensis*	TNS-F-11,932	Holo	55–3	336		
19		*superficialis*	TNS-F-12,072	Holo				
	***Cordyceps* on *Elaphomyces***							
20	*C.*	*minazukiensis*	TNS-F-197,989	Holo			*Tolypocladium* *minazukiense*	Quandt et al. 2014
21		*ophioglossides var. cuboides*	TNS-F-230,312	Holo			*T. ophioglossoides*	Quandt et al. 2014
22		*valvatistipitata*	TNS-F-230,284	Holo	46–3	369	*T. valvatistipitatum*	Quandt et al. 2014
23		×j*ezoensoide*	TNS-F-230,286	Holo				
24		*delicatistipitata*	TNS-F-230,293	Lecto	44–2	375	*T. delicatistipitatum*	Quandt et al. 2014
25		*ophioglossides f. alba*	TNS-F-18,223	Lecto			*T. ophioglossoides*	Quandt et al. 2014
	***Cordyceps* on *Cicada***							
26	*C.*	*inegoensis*	TNS-F-230,289	Holo			*T. inegoense*	Quandt et al. 2014
27		*paradoxa*	TNS-F-230,313	Holo			*T. paradoxum*	Quandt et al. 2014
28		*toriharamontana*	TNS-F-230,288	Holo			*T. toriharamontanum*	Quandt et al. 2014
29		*heteropoda*	TNS-F-230,294	Holo			*Ophiocordyceps* *heteropoda*	Sung et al. 2007
30		*longissima*	TNS-F-230,285	Holo			*O. longissima*	Sung et al. 2007
31		*prolifica var. terminalis*	TNS-F-230,295	Holo	13–2	43	*Perennicordyceps* *prolifica*	Matočec et al. 2014
32		*pseudolongissima*	TNS-F-197,983	Holo			*O. pseudolongissima*	Sung et al. 2007
33		*takaoensis*	TNS-F-3026	Holo			*O. sobolifera*	Sung et al. 2007
34		*kanzasiana*	TNS-F-198,015	Holo	11–1	37	*Polycephalomyces* *kanzashianus*	Kepler et al. 2013
35		*sinclairii*	TNS-F-212,384	Holo			*Cordyceps kobayasii*	Koval’ 1984
36		*minuta*	TNS-F-11,933	Holo				
37		*ramosipulvinata*	TNS-F-197,979	Holo	13–3	36	*Po. ramosopulvinatus*	Kepler et al. 2013
38		*ryogamimontana*	TNS-F-230,292	Holo			*Purpureocillium* *takamizusanense*	Ban et al. 2015
39		*prorifica*	TNS-F-230,300	Lecto			*Perennicordyceps* *prolifica*	Matočec et al. 2014
40		*yakusimensis*	TNS-F-230,287	Lecto	24–2	16	*O. yakusimensis*	Sung et al. 2007
41		*pleuricapitata*	TNS-F-197,965	Para				
42		*imagamiana*	TNS-F-197,966	Authentic				
43		*owariensis*	KATM-Fun01	Holo				
44		*nipponica*	KATM-Fun06, Fun 13	Authentic				
	**Other cicada pathogenic fungi**							
45	*Isaria*	*nipponica*	KATM-Fun02	Holo				
46	*I*	*oncotympanae*	KATM-Fun16-3	Holo				
47	*Synnematium*	*graptopsaltriae*	KATM-Fun03	Holo				
48	*Massospora*	sp.	KATM-Fun17	Authentic				

TNS-F: National Museum of Nature and Science; KATM-Fun: The University Museum, University of Tokyo. Data from Sato et al. (2010a, 2010b, 2012 and 2014). ^a^The colour illustration plate number in Kobayasi and Shimizu (1983). ^b^The colour illustration plate number in Shimizu (1994).

#### *Cordyceps species on* Elaphomyces

Dr Kobayasi described nine *Cordyceps* taxa (species and forma) on *Elaphomyces*, including one invalid situation that was emended by Yao et al. (1995). Four holotype specimens were discovered, and two specimens were selected as lectotypes (Sato et al. 2010b) (Table 1). One of the two was the species emended by Yao et al. (1995). Sato et al. (2010b) used the name of the genus *Elaphocordyceps* after Sung et al. (2007). The latest genus name is *Tolypocladium* (Quandt et al. 2014). Table 1 also shows the current names of each species with references.

#### Cordyceps species on Cicada

Dr Kobayasi described 20 *Cordyceps* species on Cicada, of which 19 authentic specimens, including the holotype, were discovered (Sato et al. 2012, 2014) (Table 1). From the National Museum of Nature and Science, 13 holotype specimens were discovered. Two specimens were selected as lectotypes. A paratype and an authentic specimen were also discovered (Sato et al. 2012). From the museum of the University of Tokyo, one type specimen (*C. owariensis*) and several authentic specimens of *C. nipponica* were discovered (Sato et al. 2014) (Table 1). Dr Kato’s specimens can be viewed on the web-museum of the university (http://umdb.um.u-tokyo.ac.jp/DDoubutu/katomasayo/fungi_en/index.html). Any authentic specimens of *C. polycephala*, the last one species, should be found.

At the same time, in the university museum, holotypes of *Isaria nipponica, I. oncotympanae, Synnematium graptopsaltriae* and the voucher specimen for the first record of *Massospora* in Japan (Kobayasi 1951) were also discovered (Sato et al. 2014).

## One-to-one correspondence between the type and the illustration

When the type specimens were discovered, we compared the outline of the specimens with colour illustrations in the books (Kobayasi and Shimizu 1983; Shimizu 1994). Fortunately, we recognised 22 type specimens that were illustrated in colour in the books, at present, comprising 16 cases in *Torrubiella* spp., 2 in *Cordyceps* spp. on *Elaphomyces* and 4 in *Cordyceps* spp. on cicadas (Table 1). Colour illustrations are very useful in recognising the fresh colour of the species, when the colour(s) of the types have been lost due to prolonged preservation period.

In a special case of a dried holotype specimen (*C. ramosipruvinata*), at first, we could not compare the specimen with the illustration in the books because of its shrunk stromata. Fortunately, the host morphology in the illustration, especially the traits of broken legs, was identical to those parts of the holotype specimen. The very precise illustration enabled us to associate the specimen with the illustration.

## Handling of formalin-preserved specimens

Almost all specimens were stored in formalin liquid. As cork plugs become deteriorated due to a long storage period, we must transfer each specimen into new vials. Formalin is generally a popular chemical used for preserving specimens for a long period. Its vapour has a strong poisonous odour. Fortunately, to prevent vaporisation and to maintain the level of the liquid volume, the surface of the formalin liquid was covered with an over-layer of liquid paraffin.

On the other hand, while transferring the specimens from the formalin liquid with the over-layer to new bottles, it was difficult to take out the specimen without contacting the liquid paraffin. An oil-absorbent sheet for machine maintenance has shown good results (T-151J, 3M Company). We cut the oil-absorbent sheet into small-size pieces of 1 cm × 1 cm, and put the pieces into the specimen bottles, repeating this process until the oil was completely removed. After transferring the specimen, the top of the formalin liquid in the new vials was re-covered with liquid paraffin (Sato et al. 2011).

## Ongoing survey

Specimens from Lepidoptera and Coleoptera, the major host insects of *Cordyceps*, are now being ordered. Next, the specimens of *Cordyceps* from other host groups will follow, and then, other entomopathogenic genera, including anamorphic taxa, will be the last.
